# Impact of Structural Health Monitoring on Aircraft Operating Costs by Multidisciplinary Analysis

**DOI:** 10.3390/s21206938

**Published:** 2021-10-19

**Authors:** Vincenzo Cusati, Salvatore Corcione, Vittorio Memmolo

**Affiliations:** 1 Design of Aircraft and Flight Technologies Research Group, Department of Industrial Engineering, University of Naples Federico II, Via Claudio 21, 80125 Naples, Italy; vincenzo.cusati@unina.it (V.C.); salvatore.corcione@unina.it (S.C.); 2 Aerospace Structures and Materials Laboratory, Department of Industrial Engineering, University of Naples Federico II, Via Claudio 21, 80125 Naples, Italy

**Keywords:** damage detection, aircraft structures, cost-benefit analysis, implementation strategies, multi-disciplinary analysis, direct operating cost

## Abstract

Structural health monitoring is recognized as a viable solution to increase aviation safety and decrease operating costs enabling a novel maintenance approach based on the actual condition of the airframe, mitigating operating costs induced by scheduled inspections. However, the net benefit is hardly demonstrated, and it is still unclear how the implementation of such an autonomic system can affect performance at aircraft level. To close this gap, this paper presents a systematic analysis where the impact of cost and weight of integrating permanently attached sensors—used for diagnostics- affect the main performance of the aircraft. Through a multidisciplinary aircraft analysis framework, the increment of aircraft operating empty weight is compared with the possible benefits in terms of direct operating costs to identify a breakeven point. Furthermore, the analysis allows to establish a design guideline for structural health monitoring systems returning a safer aircraft without any economic penalties. The results show that the operating costs are lower than those of the reference aircraft up to 4% increase in maximum take-off weight. Paper findings suggest to considering a condition monitoring strategy from the conceptual design stage, since it could maximize the impact of such innovative technology. However, it involves in a design of a brand-new aircraft instead of a modification of an existing one.

## 1. Introduction

Structural Health Monitoring (SHM) has been attracting the research community for the last decades in order to enable the design of lighter, safer and even cleaner aircraft. In principle, SHM is expected to avoid or reduce typical accommodations employed during design (e.g., safety knockdown factors) and lifetime management (strict scheduled inspection), inducing a cost-effective maintenance [1]. However, the industrial deployment within such a field is still limited mainly due to the concerns about the reliability assessment and the real achievable benefit in aviation. The former issue is inherent to the specific technique adopted to estimate the actual condition of the airframe and it is dealt with through a tight collaboration between experts in the domain of SHM and reliability [2]. On the other hand, the latter query requires such a multidisciplinary analysis accounting for the effects induced by SHM integration at aircraft level and including a roadmap for its efficient implementation.

Recent literature has demonstrated the effectiveness of on-condition approaches for detecting damage [3] and estimating residual useful life of aircraft [4]. Generally, this would benefit the operative costs of an aircraft being able to reduce costs related to life cycle management. This is especially true in those structures showing very complex damage mechanics, such as composites. For instance, mechanics thereof may return barely visible damage under normal loads. Namely, no exterior indentation is visible while the through thickness material is indeed compromised [5]. This is often due to the development of different mechanics which begin locally and result mostly in delamination, i.e., separation of two adjacent layers due to high interlaminar shear stresses [6,7,8]. However, impacts randomly occur on the airplane, inducing failures which may be life threatening if not addressed appropriately. This requires an opportune design, which is usually driven by the damage tolerance approach, currently either preferred to or eventually combined with safe life and fail-safe philosophies [9]. The former was used in early aviation under the concept of safety-by-retirement because component is scrapped or replaced at the end of the design life. At that time this was a viable solution because using adequate safety factors each component showed a design life comparable to or longer than the aircraft lifetime. However, it failed when fatigue issues came up and was replaced by fail-safe concept, where any fault must not endanger flight safety. This safety by design philosophy was finally encompassed by the damage tolerance approach, which involves the use of inspection procedures and structural design concepts to protect safety, rather than the traditional factors of safety used for ultimate loads [10,11,12].

In this view, the complex composite behavior is accommodated by a hard damage tolerance criterion to ensure safety in every flight condition. As accidental damage is likely to occur, it drives the design of composite structures [13]. It is generally expected that damaged components are capable to withstand operative loads without leading to any failure or enabling excessive structural deformation until the damage is detected [14]. However, the appointed damage detection approach primarily based on visual inspection does not allow for identifying damage below a certain threshold. Therefore, such an allowable damage limit is defined for different airframe parts in terms of dent depth or damage area. It is used to estimate the reduced residual strength in respect to pristine material and oversize the structure to withstand ultimate load even when damage up to that limit arises but is not detected. In addition, damage tolerance philosophy is intended to ensure that a damage equal to or larger than the prescribed threshold is reliably detected by scheduled or direct field inspections because it further reduces residual strength [15].

Impact induced damage in composite components is only one of the possible causes of airframe failures and probably that one where SHM can have the most promising impact. Indeed, many types of damage are likely to occur in commercial aircraft, such as cracks, bolt loosening, lack of bonding adhesion, and aging degradation [16]. They are commonly included in the manufacturer structural repair manual of the aircraft along with the repair plan, which mostly depends upon the damage allowable dimension and location. Hence, it is worth defining the damage size and it likely needs for nondestructive testing. As a consequence, maintenance tasks are costly because they require detailed inspection of hidden failures and a longer downtime. The result is an increasing direct operating cost (DOC) due to exceeding safety weight and safety critical operations. That is where SHM can benefit airliner costs from a conceptual standpoint. SHM aims to continuously interrogate aircraft about the structural performances of every component. Indeed, most of the damage mentioned can be monitored continuously with a variety of approaches [17]. Postulated that SHM integration can enable the detection of damage below allowable damage limit, the benefit then relies on a more flexible and faster maintenance schedule, along with an increasing level of safety.

It is worth pointing out that no assumption can be made about condition monitoring maintenance without looking into the reliability characteristics of the specific SHM system. In this view, Cottone et al. [18] applied the Bayesian damage update methodology to build up a life-cycle cost optimization model relying on reliability of aircraft components integrated with smart sensor. In particular, reliability assessment features such as probability of detection and false call rate were essential to find out the optimal inspection and replacement strategy to reduce operating costs. Such an approach has been extended in [19], where the optimization of maintenance schemes for aircraft structures is developed by investigating a small wing panel subjected to random impacts and inspected by a built-in guided wave-based multi-level SHM system. The performances are dealt by means of reliability assessment and two optimization methods, based on the minimization of the service life cost statistics. However, the discovered optimal solution is still not related to the impact returned at aircraft level. Other approaches are also available in literature to try to quantify the value of information given by SHM systems [20] or to implement a component replacement method in order to fulfil the performance requirements but also pursue a cost reduction [21,22]. However, this kind of approaches are well suited for civil engineering and rarely extended to aeronautical fields.

Although the breakthrough promised by SHM integration is within the structure and the life cycle management, the advantages remain still ambiguous and limited to an abstract conceptualization. The literature indeed shows a lack of investigations oriented to postulate the effective benefit achievable by implementing any type of structural health management. Such a discussion is indeed limited to a few examples and always related to the implementation of condition-based maintenance. Pattabhiraman et al. [23] showed that the main advantage of condition-based maintenance consists in skipping several tasks in early life when there is no severe damage detected. Using condition-based maintenance it is possible to reduce the maintenance time by 20% [24], reducing downtime and making aircraft more available [25]. In addition, Fioriti et al. [26] concluded that prognostics can improve airline profit further by making aircraft more available. However, it is increasingly important to understand how SHM-based information can be implemented in a condition-based maintenance framework.

Generally, the literature has discussed the cost-benefit of integrating real time SHM [27,28]. However, this is far from reality, being that a continuous health monitoring using onboard wireless sensors is required, as well as damage assessment using the ground station. In a more realistic approach, it is expected that efficient SHM would avoid costly inspections based on Visual and Non-Destructive Inspection (V-NDI). Detailed visual inspection is estimated to be 80–90% of the maintenance downtime. The remaining time of out of service aircraft is due to nondestructive inspection and health management, as reported in [29]. Nonetheless, the investigation carried out in that case study, encompassing the health management of the Boeing B737NG fuselage using piezoelectric transducers, reports that a large number of sensors is required to ensure similar detecting capability of classic C-check, preventing any possible benefit. In detail, the SHM system allows reducing the maintenance downtime and, as such, the maintenance cost. However, the achievable benefit is much lower than the operating cost penalty generated by the sensors system weight. Hence, it turned out that a cost-effective SHM would be achievable either improving the current sensor technologies so that fewer sensors are needed or adjusting the aircraft design concept according to SHM. However, as to the former solution, the minimum number of sensors or weight taken on board to satisfy affordability is still not clear. As to the latter possibility, Dienel et al. [30] had a further look at the SHM induced benefit, even consisting of more relaxed design constraints relying to continuous monitoring. The authors estimated a 9% weight relief achievable thanks to a guided wave based SHM system. Indeed, having a condition monitoring system implemented on-board enables the adjustment of the current damage tolerance criterion to satisfy smaller defect, which leads to structural thickness reduction. Subtracting the mass of the SHM system to that weight relief, a 5% net weight saving is finally estimated.

Despite the promising advantages, condition-based maintenance is hardly implemented in the airframe lifetime for several reasons, including the lack of accurate cost-benefit analysis, the standardization needed to comply with certification requirements and acceptance, the lack of clear requirements, and the need of tools supporting decision management [31,32,33]. Hence, although the technology is almost ready, challenges ahead are still present limiting condition-based maintenance to research and development actions [34]. As to the former criticality, the efforts made in assessing the performance of an SHM system and the advantages in integrating such a technology within airframe does not still return enough results to make a quantitative assessment of the benefits at aircraft level, demanding such an approach for the systematic quantification thereof. To fill the knowledge gap in the available literature, it is needed to assess the maximum mass that could be added to a reference aircraft to reduce operating costs without any significant performance loss. Within this context, the aim of this work is to establish a parametric study based on multi-disciplinary analysis returning the impact of the SHM system at aircraft level.

The rest of the paper is organized as follows. The methodologies adopted to calculate costs and benefit of SHM are reported along with basic concepts about the multi-disciplinary analysis approach used to estimate the impact of SHM technology at aircraft level. Then the results are showed and finally discussed before the concluding remarks are summarized in the last section.

## 2. Materials and Methods

The Materials and Methods section describes with sufficient detail the calculation of aircraft direct operating costs and aircraft flight performance needed to establish any cost/benefit at aircraft level by implementing SHM. The methodologies adopted to estimate such a performance is detailed also including a statement about aircraft structures to be monitored and the airframe SHM concepts.

### 2.1. Direct Operating Costs Methodologies

In general, each airline has its own methods for estimating operating expenses according to their operation, flight patterns, fleet, and accounting procedures. Standing the wide range of different methods and approaches, during the design process it is required to adopt a standard method for the cost estimation [35]. In a few words, the objectives of a standardized method for the estimation of aircraft operating costs are:

- To provide a means to compare aircraft designs operating costs under a specific set of conditions;
- To assist airlines and the aircraft manufacturer in assessing the aircraft economic on given routes.

Several methods are available in literature, but all derive from Standard Method of Estimating Comparative Direct Operating Costs of Turbine Powered Transport Airplanes [36], also known as ATA method, the first universally recognized set of empirical equations for estimating direct operating costs of airplanes. However, a great part of the equations comes from Association of European Airliners (AEA) method published in 1989; it is the European equivalent of the ATA method.

**Figure 1:**
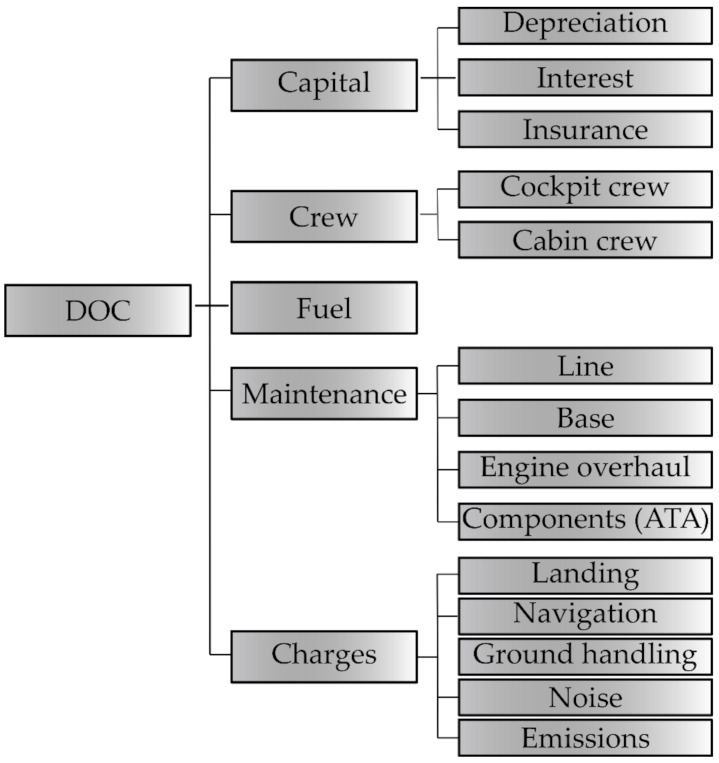
DOC breakdown.

In the following subsections the equations chosen from the methods described above for the calculation of the DOC elements are reported. As schematically illustrated in Figure 1, the direct operating cost model treated in this work includes the following items:

- Capital cost: depreciation, insurance, interest;
- Crew cost: cockpit and cabin;
- Fuel cost;
- Maintenance cost: line, base, engine overhaul, aircraft components;
- Charges: landing, navigation, ground handling, noise, emissions.

#### 2.1.1. Capital Costs

As already stated above, capital costs comprise the following financial items:

- Depreciation of the initial investment, that is the allocation of aircraft’s acquisition cost over a certain period;
- Interest charges on capital employed;
- Aircraft and passengers’ insurance.

Depreciation is the distribution of the reduction in value of an item over the useful service life. In Equation (1). TI is the total investment obtained as the sum of aircraft price and the spare costs that are that related to aircraft spare parts. The RV is the value of the aircraft at the end of the operating life. Parameters are explained in Table 1.

(1) $$DOC_{dep}\;=\;\frac{TI}{DP}\left(1-RV\right)$$

The evaluation of interest charges is a tough task since government agencies and banks can apply different charges and various fees to different customers. Charges are linked to world economic climate, local exchange rates, credits provided by national governments to airliners or to a manufacturer company to encourage the export. Equation (2) provides for a simple description of the interested cost per year, parameters are explained in Table 2. The insurance cost is linked to risks and to potential following loss. The risk of accident is well known since airworthiness authorities prescribe well assessed safety standards.

(2) $${\mathrm{DOC}}_{\mathrm{int}}\;=\;\mathrm{TI}\;\cdot {\;r}_{i}$$

The insurance company associates the probability of aircraft failure with technical risks. Together with the baseline risk there is the risk to lose an aircraft due to non-technical reasons such as terrorism [35]. In the Equation (3) Aircraft Delivery Price (ADP) is the sum of the airframe and engines prices. It is equal to TI less spares costs. Parameters are explained in Table 3.

(3) $${\mathrm{DOC}}_{\mathrm{ins}}\;=\;\mathrm{ADP}\;\cdot {\;r}_{a}$$

#### 2.1.2. Fuel Costs

For a correct estimation, it is important to first know fuel price P_fuel_ (usually given in dollars per gallon in US environment or Euro per liter in Europe) for the year in which the cost estimation is required. IATA website offers an up-to-date valuation (https://www.iata.org/en/publications/economics/fuel-monitor/ accessed on 12 May 2021). As can be noticed, fuel cost is expressed in units of volume; usually, fuel consumption per trip is given in units of mass, so it is necessary to know the jet fuel density to complete the cost estimation. Then direct operating cost associated to fuel can be the obtained by applying Equation (4). Parameters are explained in Table 4.

(4) $${\mathrm{DOC}}_{\mathrm{fuel}}\;=\;P\;\cdot {\;m}_{f}$$

#### 2.1.3. Charges: Landing, Navigation, Ground Handling, Noise, and Emissions

Landing fees are incurred for use of the airfield with its runways. Navigation charges are incurred for use of radio navigation and direction by air traffic control. Ground handling may include the following services.

In the model, the charges (DOC_charges_) are broken down into landing (DOC_ldg_), ground (DOC_grd_) and navigation (DOC_nav_) charges. The former charges can be further divided in landing (DOC_ldg_), noise (DOC_noise_), and emission (DOC_nox_) related charges as suggested in [37]. However, such differentiation can likely increase landing charges unreasonably. To deal with this drawback, a correction factor (K_eco_) is applied to accommodate the landing charges, as shown by Equation (5). This approach meets the requirements given by ICAO [37]. It is worth achieving a direct connection between noise-related charges and the basic landing fee. Thus, K_eco_ is set either to 0.635 or to 1.0, whether the noise and emission charges are considered or not [38]. The determination of landing, ground and navigation charges is based on the AEA method [39].

(5) $${\mathrm{DOC}}_{\mathrm{charges}}\;=\;K_{\mathrm{eco}}\cdot {\;\mathrm{DOC}}_{\mathrm{ldg}}{+\mathrm{DOC}}_{\mathrm{nav}}{+\mathrm{DOC}}_{\mathrm{grd}}{+\mathrm{DOC}}_{\mathrm{noise}}{+\mathrm{DOC}}_{\mathrm{emissions}}$$

**Landing charges**. It is well known that an airline must pay a fee each time an aircraft of its fleet lands at an airport. By further looking into this issue, it is interesting to find that each airport has its own taxation rules for landing charges. This fact makes difficult to create a unique model able to describe the whole regulation. As stated above, the equation proposed by AEA is here introduced. It can be noticed that landing fees generally increase with maximum take-off mass. In Equation (6). K_ldg_ is equal to 6 $/t for long range aircraft and 7.8 $/t for short-medium range airplane (MTOW is expressed in tons) [35]. Parameter explanation is reported in Table 5.

(6) $$DOC_{ldg}=MTOW\cdot K_{ldg}$$

**En-route charges**. The charges so far considered are all paid for by an airline when it enters the airspace or the ground of a specific airport. The fees treated in this section are still falling under competences of the aircraft operator but are paid to a specific authority which controls all the rest of the available airspace. In this document is analyzed the Euro- pean airspace, which is controlled by EUROCONTROL (https://www.eurocontrol.int/ accessed on 12 May 2021). Each aircraft operator which overflies EUROCONTROL Member States must pay a monthly bill. These charges are handled for EUROCONTROL by the Central Route Charges Office (CRCO) and are used to support air navigation facilities and services for safe operations. The CRCO collects four types of charges:

1. En-route charges;
2. Charges for terminal air navigation services;
3. Air navigation charges;
4. Communication charges.

Due to their specific features, air navigation, terminal, and communication charges are not modelled in this work; the implementation will concern only en-route charges. A route charge is levied for each flight performed in the EUROCONTROL airspace, which is divided into en-route charging zones; its cost is given by Equation (7). Parameter explanation is reported in Table 6.

(7) $${\mathrm{DOC}}_{\mathrm{nav}}=R\cdot K_{\mathrm{nav}}\cdot \sqrt{\frac{\mathrm{MTOW}}{50}}$$

**Ground-Handling charges**. These kinds of charges are strictly related to passengers (or amount of cargo) by nature (airport estimates these costs as a function of weight). However, each airport imposes different fees depending on the geographical position, facility dimensions and other floating parameters. Luckily, the AEA method supplies a simple formula as shown in Equation (8) to consider this part of DOC. Parameter explanation is reported in Table 7.

(8) $${\mathrm{DOC}}_{\mathrm{grd}}\;=\;\mathrm{PL}\;\cdot {\;k}_{\mathrm{grd}}$$

**Figure 2:**
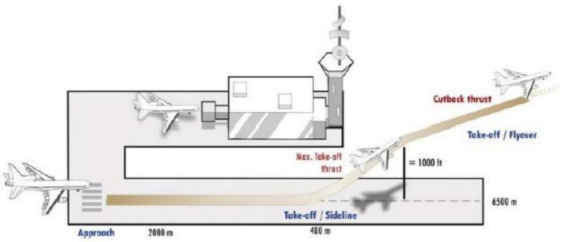
ICAO Certification Points reprinted from [40].

**Noise charges**. Since the 1970s, the ICAO has set numerous Standards for aircraft’s noise emissions that are gathered in Annex 16 of [40]. In these Standards, noise is measured in EPNdB, which is the Effective Perceived Noise level expressed in decibels. Aircraft’s noise levels are measured at three certification points, as shown in Figure 2.

Those points are identified as: (i) fly-over, located 6.5 km—measured along take-off flight path— far away from the brake release point, (ii) sideline, located 450 m far away from the runaway axis in each direction—the highest noise measured at any of those points is considered as reference value—, and (iii) approach, 2 km far away from the runaway threshold along the approach flight path.

Furthermore, the so-called cumulative levels are defined as the arithmetic sum of the certification levels at each of the three points. The responsible authorities of the various countries shall regulate noise emissions to comply the ICAO guidelines. According to the national laws, airports apply supplementary charges which increase with the noise level of the landing aircraft; this is done to encourage airlines to adopt quieter aircraft. Like landing and take-off fees, noise charges strictly depend on the airport considered. These two charge typologies are, in some cases, merged because both depend on aircraft’s weight. The methodology for noise charges calculation is the one recommended by the Transport Aircraft Noise Classification Group (TNAC) [41]; here, noise charges depend on the certified noise levels (L_approach_, L_flyover_, L_lateral_), the specific noise threshold applied to departure (T_d_) and arrival (T_a_) airport, and the unit noise rates (Δ_d_ and Δ_a_) expressed by Equations (9) and (10). When the latter rates are the same (*C_noise_*), the noise induced landing charges can be estimated by Equation (11). Parameter explanation is given in Table 8.

(9) $$\Delta_{a}\;=\;\frac{L_{\mathrm{approach}}{\;-\;T}_{a}}{10}$$

(10) $$\Delta_{d}\;=\;\frac{\frac{L_{\mathrm{flyover}}{+L}_{\mathrm{lateral}}}{2}{\;-\;T}_{a}}{10}$$

(11) $${\mathrm{DOC}}_{\mathrm{noise}}\;=\;C_{\mathrm{noise}}\;\cdot {\;(10}^{\Delta_{a}}{-10}^{\Delta_{d}})$$

**Emissions charges**. The pollutants considered in this document are NOx, CO, and HC. The term NOx is generally referred to nitrogen oxides, namely nitric oxide (NO) and nitrogen dioxide (NO_2_), which are the main responsible of air pollution; the acronym HC, on the other hand, indicates hydrocarbons, which are generated by burned or partially burned fuel and contribute to smog generation, while CO is the carbon oxide. Charges related to the emission of nitrogen oxides (NOx) per year are computed according to Equation (12). The estimation of the NOx mass emitted during Landing and Take-Off (LTO) cycle as proposed by Emission Related Charges Investigation as proposed by the Emission Related Landing Charges Investigation, which is an ECAC subgroup [42]. In similar manner, it is possible to calculate fees also for other gaseous pollutant (CO, HC, and so on). Parameter explanation is provided in Table 9.

(12) $$\begin{array}{l} {\mathrm{DOC}}_{\mathrm{NOx}}\;=\;C_{\mathrm{NOx}}\;\cdot {\;m}_{\mathrm{NOx},\;\mathrm{LTO}}\;\cdot \;a\\ \mathrm{where}:\\ \left\{\begin{array}{l} a=1\;\;\mathrm{if}\;\;\frac{m_{{\mathrm{NO}}_{X},\mathrm{LTO}}}{T}\;\leq \;19.6\\ a=\;\frac{\frac{m_{{\mathrm{NO}}_{X},\mathrm{LTO}}}{T}}{19.6}\;\;\mathrm{with}\;a_{\max}=4 \end{array}\right. \end{array}$$

#### 2.1.4. Crew Costs

Crew cost includes the salaries for the cockpit and cabin staff. The approach here applied states that crew cost can be obtained simply by multiplying a proper Labour Rate (LR) by the number of crew members (n_cm_) as shown by Equations (13) and (14). The cost calculated is quite accurate only if an exact estimation of labour rate is known. Parameter explanation is provided in Table 10.

(13) $${\mathrm{DOC}}_{\mathrm{cockpit}\;\mathrm{crew}}\;=\;{\mathrm{LR}}_{\mathrm{cockpit}}\;\cdot {\;n}_{\mathrm{cm}}$$

(14) $${\mathrm{DOC}}_{\mathrm{cabin}\;\mathrm{crew}}\;=\;{\mathrm{LR}}_{\mathrm{cabin}}\;\cdot {\;n}_{\mathrm{cm}}$$

#### 2.1.5. Maintenance Costs

The lack of such a strict definition of maintenance items makes the estimation of related costs quite challenging. As a matter of fact, the total cost assessment due to inspection, and making decisions for component replacement cannot be formulated a-priori in a general way due to the inherent variability of the mentioned actions for different aircraft and under different operating conditions.

Cost can be subdivided into Direct Maintenance Cost and maintenance burden. By definition, direct maintenance costs comprise direct airframe and engine maintenance costs; namely, those connected to material and labour demanded by maintenance actions Otherwise, maintenance burden encompasses all types of indirect costs, such as airline overhead, deprecation and maintaining costs for instrumentation, equipment and any other tools related to maintenance activities as well as costs connected to buildings and facilities to operate tasks; it is an indirect cost included in direct costs estimation. Direct maintenance cost can be evaluated in two different ways:

- by dividing maintenance in different tasks, such as line and base maintenance, engine overhaul and subsystems’ maintenance; then the cost of each activity can be estimated by itself;
- calculate the cost of labour and material for both engines and airframe maintenance.

Several methodologies which follow these approaches are available in the literature. Concerning the first approach, it is worth to mention the method proposed by Fioriti [43] which has been applied in this study since it allows for measuring the impact of a reduction in terms of line and base maintenance. Instead, regarding the second approach, there are the model presented by Harris [44], which implements the cost-estimation equations developed from the data of 67 airlines for the year 1999 referred to the Department Of Transportation (DOT). It exists also a modified version of Franz et al. of this methodology (more details can be found in [38]). Another famous methodology is that of AEA [39], which has also been used by Kundu [45] and Jenkinson [35] where the maintenance charges include labour and material costs associates with routine inspection, servicing and overhaul (for airframe, engine, avionics, systems and so on). Quite similar is the ATA method [36].

As previously stated, the first approach has been used to estimate maintenance costs, and hereafter the CERs (Cost Estimating Relationships) are presented.

(15) $$\begin{array}{r} {\mathrm{DOC}}_{\mathrm{mintenance}}=\mathrm{Line}\;\mathrm{maintenance}\;\mathrm{costs}+\\ \;\mathrm{Base}\;\mathrm{maintenance}\;\mathrm{costs}+\\ \;\mathrm{Components}\;\mathrm{costs}+\\ \;\mathrm{Engine}\;\mathrm{overhaul}\;\mathrm{costs}+\\ \;\mathrm{Burden}\;\mathrm{costs} \end{array}$$

In Equation (15), the high level costs are depicted as from reference [35]. However, in this study, the components contribution has been neglected. According to EASA Part 145, AMC 145.A.10 [46], line maintenance deals with any action undertaken before flight to let the aircraft fit for the intended flight. Instead, base maintenance copes with heavy tasks which are much more in-depth and long-lasting as less frequent than line maintenance. Significant examples are given by C- and D-checks.

In greater details, the relationships used to estimate maintenance costs are in the following Equations (16) to (20). Parameter’s explanation is provided in Table 11.

(16) DOCLine maint = 59.359 − 0.0154 ⋅ fleet size+9.9939 ⋅ U+28.325 ⋅ FHFC − 1.4008 ⋅ ageav

(17) DOCBase maint = 44.519 − 0.0116 ⋅ fleet size+7.4954 ⋅ U+21.244 ⋅ FHFC − 1.0506 ⋅ ageav

(18) $${\mathrm{DOC}}_{\mathrm{Eng}\;\mathrm{overhaul}}\;=\;135.16\;-\;19.754\;\cdot \;U\;-\;0.0189\;\cdot {\;\mathrm{age}}_{\mathrm{type}\;\mathrm{AC}}\;+11.72\;\cdot {\;N}_{e}+0.0055\;\cdot \;T$$

(19) $${\mathrm{DOC}}_{\mathrm{Burden}}\;=\;\frac{{\mathrm{DOC}}_{\mathrm{Line}\;\mathrm{maint}}+\;{\mathrm{DOC}}_{\mathrm{Base}\;\mathrm{maint}}+\;{\mathrm{DOC}}_{\mathrm{Eng}\;\mathrm{overhaul}}\;}{0.6}$$

(20) $${\mathrm{DOC}}_{\mathrm{maintenance}}{=\mathrm{DOC}}_{\mathrm{Line}\;\mathrm{maintenance}}{+\mathrm{DOC}}_{\mathrm{base}\;\mathrm{maintenance}}{+\mathrm{DOC}}_{\mathrm{Engine}\;\mathrm{ovehaul}}{+\mathrm{DOC}}_{\mathrm{Burden}}$$

### 2.2. On Condition vs. Scheduled Maintenance

Structural Health Monitoring deals with the analysis of structural performance in view of on-condition maintenance as well as integrated oriented design. However, a health management strategy relies on a complex framework where damage detection is only the first crucial action. Indeed, further critical tasks are needed once diagnosis is achieved. In addition, different stages can be identified, and likewise different methodologies can be adopted to perform those tasks [47,48,49]. Generally, the underlying concept in SHM is to record and store the structural response after a diagnostic or ambient excitation, post-process current dataset looking for features sensitive to any defect and relate such parameters to damage characteristics [50,51,52]. The core of a typical SHM system consists of transducers sparsely and permanently installed on to the structure to actuate and/or sense specific signals (e.g., vibration and ultrasound). The actuator is usually excited by a diagnostic signal. Meanwhile, the sensors record signals and data are transmitted to an acquisition system through a smart interface. In few cases the ambient excitations take the place of the diagnostic input, and the transducers are used as sensors only to record the structural response to such external loads. From this distinction, it is possible to identify active and passive SHM techniques, no matter the damage detection approach. In both cases, the current dataset needs a post-processing unit to elaborate any diagnosis. Therefore, an effective SHM system requires transducers, signal processing unit and a suited interface among them.

Using permanently distributed sensors, several approaches are available to interrogate the structure and extract features sensitive to a specific damage. They can be broadly divided in three groups: (i) vibration-based, (ii) wave propagation-based, and (iii) electromechanical impedance (EMI) techniques. The former approaches are both using the dynamic behaviour of the structure to collect useful information about its condition. The global vibration approach aims to detect presence and location of damage by analysing the frequency response functions (FRF) of the structure which is affected by damage mechanisms like delamination [52,53,54]. However, they are not effective when the defects are small compared to the dimensions of the structure. For this kind of hotspot monitoring, wave propagation or guided ultrasonic waves (GUWs) techniques are much more effective, based on the assumption that a hidden flaw in the structure locally alters the behaviour of the waves travelling in the structure. Propagation and reflection of elastic ultrasonic waves in solids [55], as well as other intrinsic GUW features [56], can be used to detect damage even in complex structures [57]. Furthermore, using multiple input (actuation) multiple output (sensing) techniques allows the overall monitoring of the component [58]. Finally, the EMI techniques exploit the electromechanical impedance response of a piezoceramic (PZT) sensor bonded or embedded into the structure to detect damage in the near field [59]. The idea behind this approach is that fault can be detected observing changes in the electromechanical signature of the sensors. However, this method is usually limited to near field damage detection, and, as such, is suitable for inspecting bolts and joints [60], or sensors themselves [48].

Other countless “non-conventional” approaches are also available and usually characterized by non-contact techniques. Among them, vision-based SHM using computer vision techniques are successfully adopted to develop accurate and low-cost bridge monitoring systems. They can identify and quantify irregular behaviours in bridge safety by simply employing single or sparse cameras and image processing techniques [61,62]. The most important advantages deal with non-contact capabilities and the wide area that may be potentially monitored. However, this cost-effective SHM system is hardly adopted for small defects detection, and it is limited to fixed targets, such as bridges and other deteriorating civil structures. A similar discussion can be organized considering global positioning system (GPS) as monitoring technology with the aim to develop a reliable and effective method of global displacement sensing [63]. However, from this point of view, both approaches may be considered as a further development of classic vibration-based techniques. Another emerging technology consists of remote interrogation of structures at microwave frequencies so that any parameter affecting the reflected wave is a function of its structural condition [64]. Damage can be detected in composite materials as well, demonstrating the feasibility of microwave sensor elements for detecting damage in composite structures. The advantages of having a damage-sensitive structure with permanently embedded sensors that can be interrogated remotely are here found together. As suggested in [65], the unique properties of millimeter wave radiation allows for penetrating through many non-conducting materials. Mechanical vibrations are rarely measured with Laser-Doppler-vibrometry (LDV) in practical SHM applications, due to the high costs. Instead, the low-cost radar technology represents a promising new approach towards in-situ SHM-scenarios with permanently installed sensors [66]. Furthermore, the low attenuation enables long distance measurements, making this radar-based approaches even an enhancement of vibration-based techniques being able to detect displacements [67] and mechanical vibration but with novel and promising capabilities [68].

From data processing standpoint, different or multiple levels of diagnosis can be provided by a SHM system according to the information collected from the signals and the specific algorithm adopted to investigate the test datasets. However, it is worth achieving both diagnosis (current condition) and prognosis (remaining useful life) to enable a condition-based lifetime strategy. In detail, the health management can be broken down in four different steps [69]: (i) damage detection, oriented to identify whether the damage is present or not according to a specific metric and decision level, (ii) damage localization, whose output allows estimating the defect location, (iii) damage quantification, which provides information about fault severity, and (iv) remaining useful life estimation, which updates the expected lifetime considering both the prescribed load history and the current damage.

This multi-level output still depends upon the condition monitoring approach envisioned. However, generally both diagnosis and prognosis output are needed. It is quite complex to provide a defined technological readiness level for such an approach, but the aim of many research and innovation projects is to move from an unproven concept (SHM system, which is standing as idea because the testing of the required technologies have been tested only) to the complete formulation of the concept, which will be optimized in terms of direct operating costs and emissions and verified by means of requirements compliance.

The SHM system development, including employed technologies and methodologies, assumes a crucial role for the enhancement of maintenance strategies of commercial aircraft. The information about aircraft operations and system condition collected by the large number of sensors can be combined with maintenance tasks carried out, logistics data and expected load scenario to achieve predictive maintenance approach effectively depending upon the health diagnostics and prognostics of the aircraft. In addition, the SHM multi-level output can feed into an adaptive health management model to let the airliner have an optimized downtime schedule and fleet management minimizing overall costs. Indeed, the implementation of SHM within maintenance strategies allows moving to an “on-condition approach” the current scheduled inspections and making scheduled the inspections unscheduled so far (due to new warning and prognosis capabilities). As a philosophy the SHM based maintenance is pro-active more than reactive, like the actual maintenance approaches. The result is expected to move from a “preventive” to a “predictive maintenance” with the aim to obtain a safer, and cheaper aircraft.

This latter goal relies on the fact that predictive maintenance, diagnostics and health monitoring aims to eliminate unscheduled groundings, eventually avoiding Aircraft On Grounds (AOGs) and the associated operational interruptions. Structural health management can tell some parts do not need an a-priori scheduled check. However, a full transition to health management will need more history, examples, and regulatory confidence so that the maintenance manual could become customized to the specific aircraft with every check based on its own operational history and current health status.

Indeed, SHM implementation within maintenance strategy requires the settling of various aspects mostly related to aircraft and damage types as well as level of safety ensured by the actual maintenance scheduling, which is a combination of different inspection categories moving from transit check to A-/B-/C-/D-checks, respectively. From the transit to the D-check, the time to perform the maintenance tasks is increasingly longer as the time interval between two following checks. The SHM system can work in place of detailed visual inspection mostly adopted in A-check and C-check (when the massive detailed visual inspection is usually up to 80–90% of AOGs time) [29]. In addition, SHM system standardization at C-check level reduces time to remove interiors, inspection costs and reduces risks of other damage while removing interiors. The remaining downtime/costs are related to NDI requested by maintenance operators for further analysis and calculation of maintenance engineers who release the aircraft or request for further repair. Hence, it can be envisioned to apply SHM at replacing visual inspection (general and detailed) and NDI (much more expensive) levels while engineers continue working as maintenance supervisors (for release/repair). Hence, a huge amount of costs is reduced positioning SHM in place of transit- and A-checks, limiting inspection according to SHM warning, and strongly simplifying the C-check.

To feed the MDA implemented hereinafter, an exemplary SHM system is considered to achieve realistic results, but without prejudicing the generality of the methodological framework. This latter is independent of the SHM system adopted as further discussed in Results section. The considered system relies on the use of small lead zirconate titanate (PZT) transducers, which are light (about 2 g) and effective for GW or EMI interrogation. The current technology is well suited for cabling and requires an analog to digital converter device together with a switch system and a power amplifier to drive the PZT interrogation. The power electronics are available on the market and customizable into small boards capable to interface with up to 25 sensors [70], and a weight around the kilos. In addition to that, cables and connectors are needed, whose weight is estimated around 7.5 g/m and 2 g, respectively. The whole system placed on-board is then supposed to be connected to a workstation for SHM inspection on the ground or eventually to the aircraft control unit. Summing up weight and cost of the equipment, it is then possible to get to the standard weight and cost per sensor unit, as further discussed in the Results section.

### 2.3. Aircraft Performance Estimation

**Figure 3:**
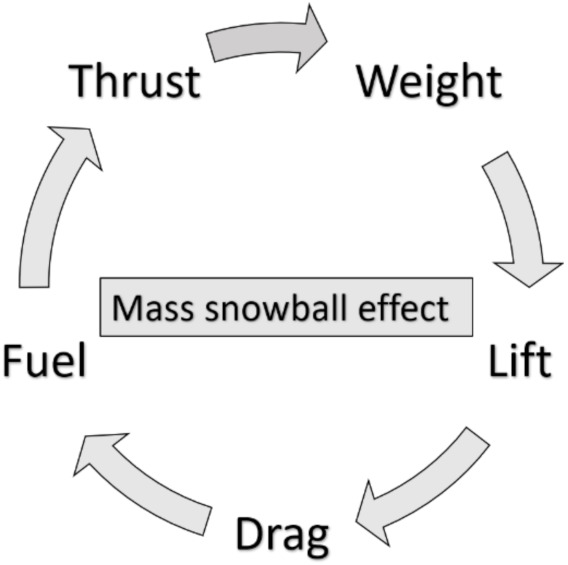
Representation of the mass snowball effect.

To provide a reliable estimation of possible benefits brought by SHM the impact such a technology has on aircraft performance must be well assessed at aircraft level. The introduction of permanently attached sensors to aircraft structures affects the mass of each of the aircraft components touched by this technology. At aircraft level the impact that a mass variation of a single component has is not linear according to the so-called snowball effect illustrated in Figure 3, where the payload is assumed to be constant.

If the weight of structures or systems is increased an increase of the total aircraft weight is expected. Leading to the following:

- greater required lift;
- larger wing;
- higher aerodynamic drag;
- therefore, the thrust must be increased;
- this leads to larger engines;
- this increases the weight again.

This process is iterative and continues until the weight converges. Standing the above, the effects of extra masses introduced by SHM system must be well investigated at aircraft level by means of a Multi-Disciplinary-Analysis (MDA) framework. This will allow to provide a reliable estimate of advantages and drawbacks of applying a SHM technology.

**Figure 4:**
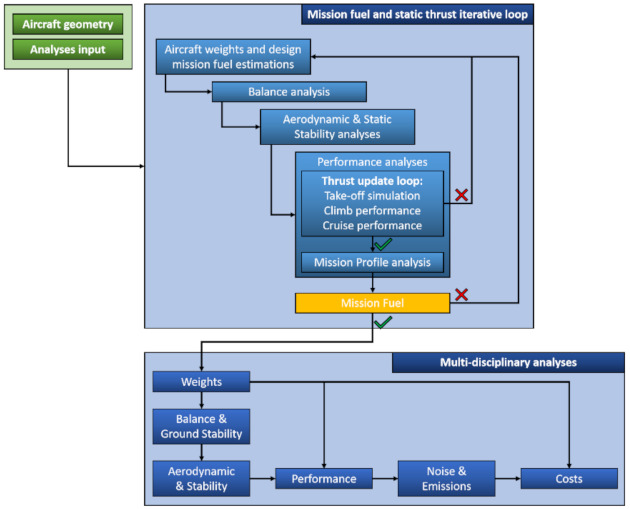
JPAD MDA workflow including thrust update inner loop. Reprinted from [74].

An MDA workflow couples the major aircraft design disciplines such as weight, balance, aerodynamics, performance, and DOC. Figure 4 illustrates the MDA cycle, this latter is accomplished though JPAD (Java toolchain of Programs for Aircraft Design). JPAD is a software developed at the Industrial Engineering Department of the University of Naples Federico II [71,72,73].

The MDA loop begins with a well-educated first guess of the fuel required to fulfill a certain mission. To estimate the center of gravity excursion range, the balance analysis is carried out. Then, aerodynamic and stability modules perform the estimation of the trimmed aircraft lift and drag polar curves for each of the estimated center of gravity position. The last step of the MDA loop deals with the performance estimation through a simulation-based approach to calculate the new amount of fuel required to cover the specified mission range. Finally, an iterative loop is fulfilled to estimate the right fuel mass (and thus the aircraft maximum take-off weight) required for the selected mission profile. The loop converges when the first estimated fuel mass equals the one calculated at the mission profile analysis stage. Within the iterative loop to calculate the fuel mass illustrated in Figure 4, there exists the possibility to trigger a second nested iteration loop. This loop can be performed to verify if the aircraft ground performance (i.e., take-off field length and landing field length) and the maximum cruise speed (in terms of Mach number) are compliant with aircraft requirements. To adjust cruise and on-ground performance, within the second nested iterative the reference static thrust of all the engines can be scaled. By scaling the reference thrust the engine weight can be updated as well.

Mass estimation of weights and balance of each aircraft component and onboard systems are performed using the formulations and approaches suggested by Torenbeek in [75].

Since this work seeks to highlight the net effects at aircraft level of the introduction of SHM technology, the abovementioned second convergence loop, concerning the thrust scaling and the engine mass updating, will be not performed. This allows for evaluating the negative effects on aircraft performance due to the additional mass introduced by the application of permanently attached monitoring sensors.

## 3. Results

The main motivation behind this research work is to measure the possible reduction of DOC by implementing SHM. References discussed in Section 1 estimate the increment of the weight due to sensors which constitute SHM system, and then try to understand what the new direct operating costs could be. Conversely to this work, they generally neglect the mass snowball effect (described in Section 2.2) and how this will impact on the aircraft maximum take-off weight and, in turn, how the higher MTOW will affect aircraft performance. Through a multidisciplinary analysis framework, the increment of aircraft operating empty weight can be compared with the possible benefits in terms of direct operating costs. Nonetheless, neither the SHM system mass that can be added to the airframe without any cost penalties, nor the number of sensors needed to reduce the maintenance costs are well known so far. Thus, it is of utmost importance to address this investigation through a parametric study, assuming the sensor density as input variable of the SHM system. This procedure allows to identify the breakeven point between the aircraft MTOW (increased by sensors’ mass) and the variation in aircraft DOC (mainly modified by the maintenance costs and sensor integration). Indeed, that density is even representative of the technological level that the SHM system consists of. Therefore, this analysis is valuable to derive the cost trend-lines over the number of sensors adopted without making assumption on the SHM technological level.

To evaluate the net possible benefits introduced by SHM technology on the DOC, this work proposes a parametric study by changing the density of SHM sensors in terms of number of sensors per square meter. The idea is to select an existing flying platform and to apply a number of health monitoring sensors to main components structures (i.e., wing, fuselage and empennage). The application of SHM sensors will introduce an extra mass triggering the mass snowball effect (see Figure 3). Previous research works faced this aspect by reducing the number of passengers (or the payload) to keep constant the maximum takeoff weight avoiding, this way, the resizing of aircraft structures and assuming that the aircraft performance is unchanged (except for the reduced number of passengers).

Clearly, by aircraft design point of view, the introduction of SHM technology means to fully re-design the aircraft to preserve main performance like field lengths, time to climb, block fuel, block time, emissions, etc. The inclusion of SHM technology should be approached already at the early stages of aircraft design. To get the most out of such a technology, a multidisciplinary optimization should be accomplished at aircraft level to define the optimum aircraft solution compliant with a specific set of top-level requirements aiming at the minimum DOC or at the minimum environmental impact.

This work is meant to be a first step towards the inclusion of the SHM technology at the early stages of aircraft design. The analyses here presented want to provide useful indications to quantify possible benefits in terms of DOC, brought by the adoption of SHM, and evaluating, at the same time, the detrimental effects of an increased aircraft weight in terms of performance without changing the target payload and mission profile.

Since it is not possible to quantify the amount of SHM sensors needed to reduce the aircraft maintenance without getting together many management aspects and details about the methodology adopted for condition monitoring, the parametric investigation proposed in this work intends to lay out a reference design framework to conceive cost effective SHM systems. Indeed, the investigation is performed by changing the number of sensors per square meter applied on aircraft main components. That is to say, the results can be exploited and applied to any SHM system to predict the impact on the aircraft costs by assuming the actual sensor density (or mass) needed to ensure a certain maintenance benefit. Hence, in such an inverse approach, the SHM designer can verify the suitability and affordability of the system conceived simply by computing the weight penalty introduced. In other words, the aim is to define a break-even point between the aircraft mass increment and the reduction of DOC. At the same time, the investigation provides information about the aircraft performance degradation in order to evaluate if the loss in aircraft performance is worth the benefits in terms of DOC.

Targeting the minimum impact into re-designing the aircraft embedding the SHM technology, the multidisciplinary investigation has been accomplished under two main assumptions. The first assumption consists of keeping constant the external dimensions of the aircraft. This means to keep unchanged the geometrical parameters of aircraft components (i.e., wing area, wing aspect ratio, empennage sizes, etc.). The other assumption consists of keeping constant the installed power (the power plant remains unchanged). In particular, the cost of engine replacement would have an impact such that to cancel all possible benefits introduced by SHM.

The jet aircraft selected as reference platform with respect to which estimate the effects of SHM technology is similar to the Airbus A220-300. The reference aircraft is modeled through JPAD software assuming the set of TLARs of the Airbus A220-300. All the required data of the reference aircraft were retrieved from public sources such as the aircraft manual, the European Aviation Safety Agency (EASA) data sheets, public data archives, and the Base of Aircraft Data (BADA) database by looking at A220-300 like aircraft models [76,77,78,79,80]. The main aircraft characteristics are summarized in Table 12.

Thanks to the available data and through a digitization process of the A320-300 three views, the parametric model of the reference platform was prepared. This digital model was used to perform the multi-disciplinary analysis cycle (neglecting the static thrust update loop but including the mission fuel feedback loop) in order to trim the analysis tool to match the A320-300 performance and benchmarking the reliability of the analyses that can be carried out by means of JPAD software.

To estimate aircraft DOC, the fuel price was assumed according to IATA fuel price monitor [81], and the aircraft price was estimated by considering the available reference [82]. The unit costs of the engine were estimated from data available in [83].

To validate the calculation case, Table 13 compares the JPAD output with publicly available data dealing with the A320-300 (see Table 12). In Table 13, the good agreement with the available data and the estimated ones can be appreciated.

Once the reliability of the MDA tools has been validated, the same software setup has been used to perform a parametric study about different values of the weights, coming from a different value of sensor density defined as number of sensors per square meters. In this work, an equal density distribution has been assumed on each aircraft component, which values vary from 0 (aircraft without SHM) to 50 sensors per square meter.

Furthermore, for each sensor density value, the costs burden and weight increment at aircraft level were estimated as well. As concerns the aircraft performance, the authors choice was to monitor the main characteristics without imposing constraints since the basic idea is to analyze the impact on an existing aircraft, to find out what should be the cost benefit due to the SHM system rather than to design a new aircraft optimized for maintenance aspects.

The proposed methodology for DOC turns out to be crucial in the early stage of a new aircraft program. In this respect, the aim of the research here presented is to offer a fast and reliable tool for considering cost related issues since the conceptual design phase.

However, to perform a realistic estimation of the DOC, it is necessary to make several assumptions regarding peculiar parameters. As already stated, the reference aircraft is a jet aircraft similar to the Airbus A220-300. Starting from the capital costs, it is necessary to estimate the aircraft and the engine prices. In this case, the price has been estimated starting from the real price of the Airbus A220-300 plus the cost of SHM sensors (considering a return factor of 10% due to innovative aircraft).

According to the technological level of the SHM solution, the sensor price is only a minimum part of the system cost. As mentioned above, to have a PZT based SHM system, it is indeed necessary to equip the whole aircraft with a wiring system (including wires and connectors) and a specific compact power electronics hardware (including a control board, a smart processor on chip and analog to digital converters) needed to enable sensor control and allow signal acquisition Considering the current market prizes, each sensor unit requires a dedicated local system whose cost is assumed as 230$ per piezoelectric transducer when PZT-based actuation and sensing SHM system is considered. In addition, there is an una-tantum integration cost along with a possible concurrent cost for the system management. The former is due to the cost of the system installation. This slightly increases the aircraft manufacturing costs but it can be reasonably included within the overall costs for aircraft systems integration. Instead, the latter is due to the maintenance of the system itself and it is included within the maintenance costs of the aircraft.

In addition to the direct cost of the equipment, it is worth noting that each component moved on board has its own weight, which has been also characterized according to the considered SHM solution. Sensors, wires, and connectors along with hardware needed for multi-channel actuation and acquisition lead to a weight penalty of about 80 g per transducer. That mass returns a sort of cost penalty which has been underestimated in many cost-benefit estimations so far, where a certain cost per flight hour induced by the added mass is considered without accounting any effect on aircraft performance [84].

Strictly related to prices is the spare cost, which could be defined as the sum of the cost of the engine and aircraft spare parts. Usually, the cost of aircraft spare parts is assumed as the 10% of the aircraft cost, while the engine spare part as the 30% of the engine cost [35]. The Total Investment (TI) is the sum of the aircraft price and the spare costs.

**Figure 5:**
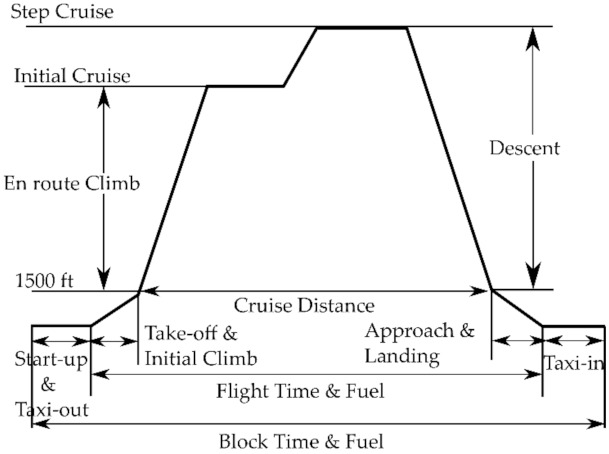
Mission profile as defined by Association of European Airliners [85].

Another fundamental parameter is the Utilization (U), defined as the number of revenue hours per year (revenue hours is time associated with the block time and does not include training, positioning for schedule, or any other non-revenue flying). The Block Time (BT) is the total time spent from starting engines to engines off, while the Flight Time (FT) is equal to BT less ground maneuver time. The relationships between BT and FT, and the correlation for U is suggested by AEA [85]. The scheme shown in Figure 5 helps to make clear the definitions just introduced.

All DOC items could be expressed in several ways based on the Utilization (U), Block Time (BT), and number of passengers. Each of them could give different information on the aircraft operation’s impact. In fact, the operating cost could be reported as hourly cost in US$/h or in a route cost obtained by multiplying the hourly DOC by the BT (US$/flight).

This latter parameter can be divided by the block distance to get to the mile cost (US$/NM) and then by the maximum number of seats to define the seat mile cost ((US$)/(NM seat)). To have a further look into those costs, some manufactures quote them on cash basis by removing the capital costs from the total DOC. This is because airliners commonly lease the aircraft yearly and the related cost is rather accounted within the company balance sheet separately

To make clearer and summarize what is stated above, Table 14 and Table 15 highlights respectively the economic assumptions, weights and performance data used for the comparison.

**Figure 6:**
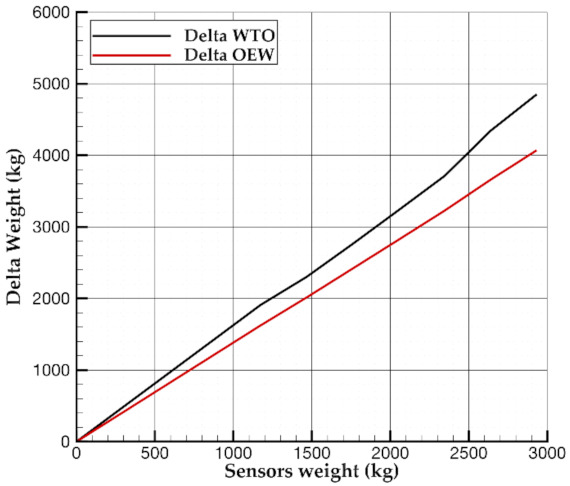
Effect of sensors weight on aircraft WTO and OEW.

Results shown in Table 16 and Table 17 and Figure 6 represent the core of the present research work, summarizing the main findings of the parametric investigation carried out. The tables mainly report the effects of sensors (whose metric relies on the density per airframe square meter) on weight and aircraft DOC and flight performance. The former shows the impact of the sensors weight on the aircraft Operating Empty Weight and Maximum Take-off Weight. Those results highlight how the extra mass introduced by SHM sensors does not apply linearly to aircraft OEW or WTO. This effect is graphically shown in Figure 6, where the increments of aircraft OEW and WTO are plotted versus the SHM weight. This chart clearly highlights that to 1 kg of SHM do not correspond 1 kg of additional mass of OEW or WTO. This latter highlights how the aircraft mass snowball effect impacts on the estimation of the DOC net benefits due to the SHM. To have a further look into the weight change, Table 16 also shows how every aircraft component mass change by assuming different SHM sensors densities. Increments are also provided in terms of percentage variations with respect to the reference aircraft represented by the first raw in the table. Table 17 summarizes the aircraft performance and DOC against the density of sensors adopted. It is worth noting how the DOC can be either lower or higher than that of the reference aircraft. Instead, the remaining parameters are generally penalized by the increased mass of the aircraft.

Since the maintenance cost is the sum of line, base, and engine overhaul (components costs are not considered in the present work), to evaluate the impact of the SHM technology on maintenance costs, an assumption is needed. Results discussed in this work are achieved by assuming that SHM allows for reducing the costs as follows:

- 50% of line maintenance since it should not be necessary to perform any preventive actions thanks to the information gathered through the sensors;
- 50% of base maintenance since it seems unrealistic the hypothesis for which it is possible to eliminate completely the so-called Check C (from regulation point of view).

**Figure 7:**
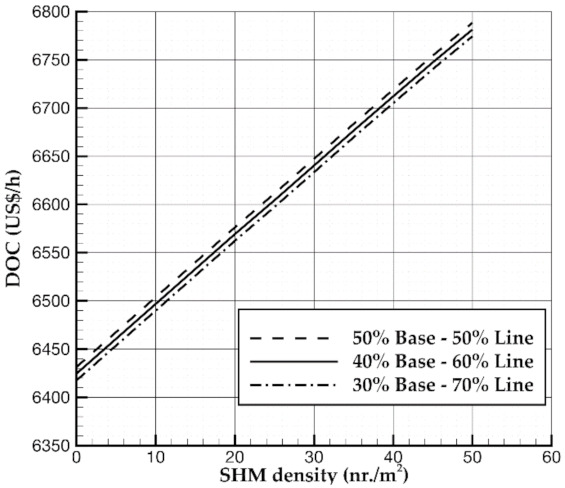
DOC variation with different assumptions of the impact of SHM on base and line maintenance.

Anyway, this assumption has an almost negligible effect on the DOC estimation. The authors have performed analyses with other two assumptions: 40–60% and 30–70% of reduction of base and line maintenance respectively. Results illustrated in Figure 7 clearly show how the assumption made to estimate the SHM impact on base and line maintenance is of a little interest because the effect on DOC is negligible. Nonetheless, the findings highlight that SHM systems focused to reduce line maintenance as much as possible can lead to greater benefits.

It is worth noting that to achieve the assumed reduction of the maintenance cost, the number of required sensors per square meter is unknown. This value relies on the specific methodology adopted to detect damage and apply health management. That is to say, it reflects the technological level of the SHM systems and may vary according to the innovation thereof. However, given the assumptions about cost and weight induced by sensor integration according to the density all through the airframe, it is possible to get to a quantitative information about the induced DOC. Indeed, these trends provide an aseptic quantification of the resulting operating costs according to the number of sensors placed on the aircraft and provide a first guideline to design an affordable SHM system. The same discussion can be undertaken considering the operative empty weight induced by sensor integration and DOC variation.

**Figure 8:**
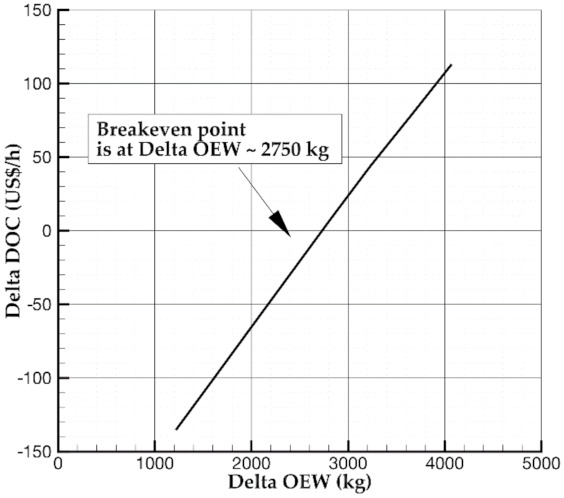
Breakeven point for the reference aircraft: Delta DOC vs. Delta OEW due to SHM.

**Figure 9:**
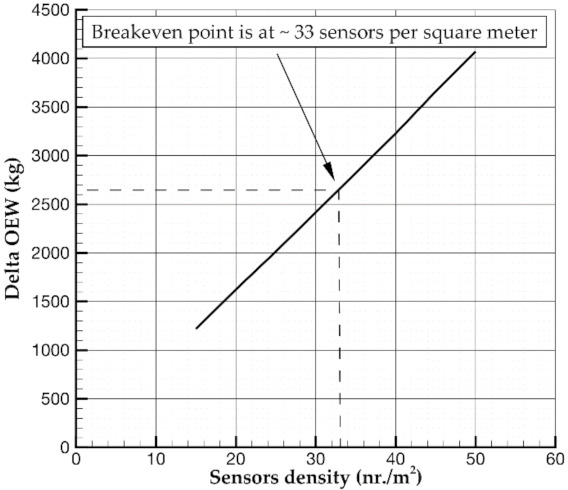
Breakeven point estimation for the reference aircraft: Delta OEW vs sensors density.

Figure 8 shows the difference in operating costs between reference and SHM-equipped aircraft versus the increase in the operative empty weight. In detail, a negative value means that costs reduce. Otherwise, a positive variation relies on a DOC increase. In particular, the breakeven point position demonstrates that SHM integration could lead to lower operating costs than those of a reference aircraft till 4% MTOW increment brought by the introduction of the SHM system. To have further insight into the SHM design, Figure 9 reveals the break-even point in terms of sensor density. According to the assumption made about the SHM system and mass and costs of several components, the use of 33 sensors per square meter returns a DOC close to the reference aircraft. The advantage still relies on the safety increase provided by the SHM system.

To summarize the achieved results, it is worth highlighting once again that the parametric trends are drawn regardless of the SHM technological level. Hence, the break-even point returns the upper limit for the weight induced by the SHM system. That is to say, the mass of the new system that is integrated within the aircraft should not exceed the mass increment at the break-even point to prevent significant performance loss. This result is independent of the system type, laying the foundation for a new way to design the SHM system. So far, it is hardly recognized a-priori the value of the latter at aircraft level. Given this insight, the SHM designer already knows the weight constraints to get to an affordable system in terms of direct operating costs of the aircraft. Hence, it is a-priori well-known whether implementing SHM is convenient or not while conceiving a specific SHM solution. In light of this discussion, sensor density becomes the principal SHM parameter to be formulated to estimate costs induced by the system and understand the value of the system in terms of costs. Just to give an impression, Table 18 shows the link between density and estimated costs per components. Having a closer look at the results, the impact of fuselage is of paramount importance because it induces more than 50% of the added weight. According to the specific SHM technological level, this suggests that an interesting solution could rely on designing the SHM system with variable sensor density to achieve the best compromise between costs and benefits.

Although the break-even point suggests a promising impact of the SHM at aircraft level, it is worth noting that around that value, the aircraft performance is relatively compromised, as reported in Table 17. Despite that a safer aircraft at the same operating costs could be achieved by integrating SHM, for example the longer take-off field length strongly limits the airport access making the aircraft less attractive for the airliners. In addition, the fuel consumption increases with a critical impact on the aviation footprint in contrast to the clean aviation paradigms.

This paper is intended as a first effort to look into the evaluation of the effective benefits brought by the adoption of SHM technology. Nonetheless, the integration of SHM is something that needs to be considered since the early stages of aircraft design in order to develop new aircraft that could maximize the potential benefits of such a technology.

## 4. Conclusions

The introduction of SHM technology enables to introduce an on-demand screening of the current airframe health increasing flight safety during the whole aircraft lifetime. However, integrating SHM systems demands the aircraft re-design to preserve main performance like field lengths, time to climb, block fuel, block time, emissions, etc. This paper approaches the problem through a multidisciplinary analysis at aircraft level to provide useful indications to quantify possible benefits in terms of DOC, brought by the adoption of SHM. The parametric analysis carried out on a A220-like aircraft shows that the operating costs of SHM-equipped aircraft could be lower than those of a reference aircraft till 4% MTOW increment brought by the introduction of the SHM system. In addition, the parametric trends are drawn regardless the SHM technological level. Hence, the break-even point returns the upper limit for the SHM system weight. That is to say, the mass of the new system that is integrated within the aircraft should not exceed the mass increment at the break-even point to prevent significant performance loss.

This paper wants to be a first effort in investigating the net potential benefits from the implementation of a SHM technology. However, the integration of SHM is an aspect that must be considered since the early stages of aircraft design to develop new aircraft that could maximize the potential benefits of such a technology.

## Figures and Tables

**Table 1:** Depreciation symbols explanation.

Symbol	Explanation
DP	Depreciation Period (years)
TI	Total Investment
RV	Residual Value
DOC_dep_	DOC of depreciation per year

**Table 2:** Interest cost symbols explanation.

Symbol	Explanation
TI	Total Investment
r_i_	Annual rate
DOC_int_	DOC of interest per year

**Table 3:** Insurance cost symbols explanation.

Symbol	Explanation
ADP	Aircraft Delivery Price
r_a_	Annual rate
DOC_ins_	DOC of insurance per year

**Table 4:** Fuel cost symbols explanation.

Symbol	Explanation
P_fuel_	Fuel Price
m_f_	Fuel Mass
DOC_fuel_	DOC fuel

**Table 5:** Landing cost symbols explanation.

Symbol	Explanation
K_ldg_	Unit rate (US$/t) equal to 7.8 for short-medium range and to 6 for long range
MTOW	Maximum Take-Off Weight
DOC_ldg_	DOC landing

**Table 6:** Navigation cost symbols explanation.

Symbol	Explanation
K_nav_	Unit rate (US$/km∗$\sqrt{t}$) equal to 0.5 for short-medium range and to 0.17 for long range *
$\sqrt{\frac{MTOW}{50}}$	Distance factor
R	Range (km)
DOC_nav_	DOC related to en-route navigation charge

* As suggested by the AEA method [39].

**Table 7:** Ground handling cost symbols explanation.

Symbol	Explanation
K_grd_	Unit rate (US$/t) equal to 100 for short-medium range and 103 for long range *
PL	Payload
DOC_grd_	DOC related to ground-handling charges

* As suggested by the AEA method [39].

**Table 8:** Noise cost symbols explanation.

Symbol	Explanation
C_noise_	Unit noise rate ($)
L_approach_	Certified noise level—approach measure point (EPNdB)
L_flyover_	Certified noise level—approach measure point (EPNdB)
L_lateral_	Certified noise level—lateral measure point (EPNdB)
T_d_	departure airport threshold noise (EPNdB)
T_a_	arrival airport threshold noise (EPNdB)
DOC_noise_	DOC related to noise emissions

**Table 9:** Emission cost symbols explanation.

Symbol	Explanation
C_NOx_	Unit rate (US$) for NOx
m_NOx,LTO_	mass of NOx emitted during LTO kg
DOC_NOx_	DOC related to NOx emissions

**Table 10:** Crew cost symbols explanation.

Symbol	Explanation
LR	Labour Rate
n_cm_	Number of crew member

**Table 11:** Maintenance cost symbols explanation.

Symbol	Explanation
U	Utilization (h/day)
FH	Flight hour
FC	Flight cycle
${\mathrm{age}}_{\mathrm{av}}$	Aircraft average age (years)
${\mathrm{age}}_{\mathrm{typeAC}}$	Age of type of aircraft (months)
$N_{e}$	Number of engines
T	Thrust (lbf)
${\mathrm{DOC}}_{\mathrm{Line}\;\mathrm{maint}}$	DOC related to Line maintenance
${\mathrm{DOC}}_{\mathrm{Base}\;\mathrm{maint}}$	DOC related to Base maintenance
${\mathrm{DOC}}_{\mathrm{Eng}\;\mathrm{overhaul}}$	DOC related to Engine overhaul
${\mathrm{DOC}}_{\mathrm{Burden}}$	DOC related to Burden
${\mathrm{DOC}}_{\mathrm{maintenance}}$	DOC related to total direct maintenance

**Table 12:** Main data concerning the A220-300, derived from [79].

TLAR	
Accommodation (Typical-Full Economy)	135
Design range (typical)	3100 NM
Take-Off Field Length (Max Take-Off Weight, ISA conditions, Sea Level)	1890 m
Landing Field Length (Max Take-Off Weight, ISA conditions, Sea Level)	1509 m
Cruise Mach number (typical)	0.78–0.80
Cruise altitude (typical)	37,000 ft
Max cruise Mach number at 37,000 ft	0.82
Max operating altitude	41,000 ft
Alternate cruise range (assumed by authors)	200 NM
Alternate cruise altitude (assumed by authors)	20,000 ft
Holding duration (assumed by authors)	30 min
Holding altitude (assumed by authors)	1500 ft/min
Residual fuel reserve (assumed by authors)	5%
**Geometrical and Operational Data**	
Wing area	112.3 m^2^
Wingspan	35.1 m
Wing aspect ratio	10.97
Fuselage length	38.71 m
Fuselage diameter	3.7 m
Single engine static thrust	24,400 lbf
Engine by-pass ratio	12:1
Max Take-Off Weight	67,585 kg
Max Landing Weight	58,740 kg
Max Zero-Fuel Weight	55,792 kg
Operating Empty Weight	37,081 kg
Max Payload	18,711 kg
Max Fuel Mass	17,726 kg
BADA averaged climb speed (CAS)	271 kt
BADA averaged rate of climb	1642 ft/min
BADA maximum rate of climb	2862 ft/min
BADA averaged descent speed (CAS)	218 kt
BADA averaged rate of descent	2186 ft/min
BADA maximum rate of descent	3700 ft/min

**Table 13:** Comparison between JPAD output and A220-330 data in Table 12, updated from [74].

Parameters	JPAD	A220-300	Difference (%)
Max Take-Off Weight (kg)	66,956	67,585	−0.93%
Max Landing Weight (kg)	56,875	58,740	−3.18%
Max fuel Mass (kg)	17,553	17,726	−0.98%
Max Zero-Fuel Weight (kg)	53,951	55,792	−3.30%
Operating Empty Weight (kg)	36,916	37,081	−0.45%
Take-Off Field Length (m)	1837	1890	−2.78%
Landing Field Length (m)	1509	1509	0.00%

**Table 14:** Economic assumptions.

Life span	16	years
Residual value	10%	
No. seats	135	
Aircraft price	101.8	US$ million
Engine price (each)	12	US$ million
Spares	14.9	US$ million
Interest	5.4%	per year
Insurance	0.5%	per year
No. of flights	558	
Utilization	3750	h/year
Block Time	6.72	h
Block Fuel (mission)	14,402	kg
Age of type of aircraft	24	months
Average age	1	years
Fleet size	30	
Fuel Price	1.4	US$/gal

**Table 15:** Data for DOC estimation: reference mission data and weights.

Performance 16 Years		
Range	3100 NM	
Mach cruise	~0.80	
SFC (Specific Fuel Consumption at cruise)	0.532	lb/(lb ∗ h)
T_0_ (thrust)	24,400	lb
**Weights**		
MTOW	66,956	kg
OEW	36,916	kg
PAYLOAD	14,648	kg
FUEL (mission)	15,393	kg

**Table 16:** Weight estimation for different component for each sensor density.

Density (nr./m^2^)	Sensors Weight (kg)	OEW (kg)	MTOW (kg)	Fuselage Weight (kg)	Wing Weight (kg)	H-Tail Weight (kg)	V-Tail Weight (kg)
0	0	36,916	66,956	7101	6880	812	653
15	880	38,134 (+3%)	68,388 (+2%)	7580 (+7%)	7265 (+6%)	880 (+8%)	710 (+9%)
20	1173	38,540 (+4%)	68,866 (+3%)	7740 (+9%)	7393 (+7%)	903 (+11%)	728 (+11%)
25	1466	38,930 (+5%)	69,257 (+3%)	7899 (+11%)	7519 (+9%)	926 (+14%)	747 (+14%)
30	1759	39,333 (+7%)	69,717 (+4%)	8059 (+13%)	7647 (+11%)	949 (+17%)	766 (+17%)
35	2053	39,738 (+8%)	70,191 (+5%)	8218 (+16%)	7775 (+13%)	972 (+20%)	784 (+20%)
40	2346	40,144 (+9%)	70,669 (+6%)	8378 (+18%)	7902 (+15%)	995 (+23%)	803 (+23%)
45	2639	40,576 (+10%)	71,300 (+6%)	8537 (+20%)	8032 (+17%)	1018 (+25%)	822 (+26%)
50	2932	40,986 (+11%)	71,807 (+7%)	8697 (+22%)	8160 (+19%)	1040 (+28%)	841 (+29%)

**Table 17:** Aircraft performance and DOC at different sensors density for the design mission (see Table 12).

Density (nr./m^2^)	DOC (US$/h)	TO Field Length (m)	Time to Climb (min)	M Cruise	Landing Distance (m)	Block Fuel (kg)	Block Time (min)
0	6675.2	1837	17.38	0.80	1509	13,706	401
15	6540.0 (−2.03%)	1912 (+4%)	18.22 (+5%)	0.79 (−1%)	1516 (+1%)	13,897 (+1%)	400 (+0.1%)
20	6576.2 (−1.48%)	1938 (+5%)	18.51 (+7%)	0.79 (−1%)	1518 (+1%)	13,961 (+2%)	400 (+0.1%)
25	6611.3 (−0.96%)	1958 (+7%)	18.76 (+8%)	0.79 (−1%)	1519 (+1%)	14,011 (+2%)	400 (+0.1%)
30	6647.3 (−0.42%)	1983 (+8%)	19.06 (+10%)	0.78 (−2%)	1521 (+1%)	14,073 (+3%)	400 (+0.2%)
35	6683.6 (+0.12%)	2009 (+9%)	19.37 (+11%)	0.78 (−2%)	1523 (+1%)	14,137 (+3%)	400 (+0.2%)
40	6719.1 (+0.66%)	2035 (+11%)	19.70 (+13%)	0.78 (−2%)	1526 (+1%)	14,207 (+4%)	400 (+0.1%)
45	6754.4 (+1.19%)	2070 (+13%)	20.14 (+16%)	0.77 (−3%)	1531 (+1%)	14,315 (+4%)	402 (+0.3%)
50	6788.4 (+1.70%)	2099 (+14%)	20.51 (+18%)	0.77 (−3%)	1534 (+2%)	14,402 (+5%)	403 (+0.7%)

**Table 18:** SHM system characteristics.

Component	Density (nr./m^2^)	Estimated Costs (€)	Weight (kg)
Fuselage	50	4,476,163	1596
Wing	50	2,582,084	921
Horizontal tail	50	641,506	229
Vertical tail	50	524,632	187

## Data Availability

The data presented in this study are available on request from the corresponding author.
